# New Organosilicon Composite Based on Borosiloxane and Zinc Oxide Nanoparticles Inhibits Bacterial Growth, but Does Not Have a Toxic Effect on the Development of Animal Eukaryotic Cells

**DOI:** 10.3390/ma14216281

**Published:** 2021-10-21

**Authors:** Denis N. Chausov, Dmitriy E. Burmistrov, Alexander D. Kurilov, Nikolai F. Bunkin, Maxim E. Astashev, Alexander V. Simakin, Maria V. Vedunova, Sergey V. Gudkov

**Affiliations:** 1 Prokhorov General Physics Institute of the Russian Academy of Sciences, Vavilova St. 38, 119991 Moscow, Russia; d.chausov@yandex.ru (D.N.C.); dmitriiburmistroff@gmail.com (D.E.B.); ad.kurilov@gmail.com (A.D.K.); nbunkin@mail.ru (N.F.B.); astashev@yandex.ru (M.E.A.); avsimakin@gmail.com (A.V.S.); MVedunova@yandex.ru (M.V.V.); 2 Bauman Moscow State Technical University, Vtoraya Baumanskaya ul. 5, 105005 Moscow, Russia; 3 Institute of Biology and Biomedicine, Lobachevsky State, University of Nizhni Novgorod, 23 Gagarin Ave., 603950 Nizhny Novgorod, Russia

**Keywords:** ZnO nanoparticles, borosiloxane, antibacterial agents, composite materials

## Abstract

The present study a comprehensive analysis of the antibacterial properties of a composite material based on borosiloxane and zinc oxide nanoparticles (ZnO NPs). The effect of the polymer matrix and ZnO NPs on the generation of reactive oxygen species, hydroxyl radicals, and long-lived oxidized forms of biomolecules has been studied. All variants of the composites significantly inhibited the division of *E. coli* bacteria and caused them to detach from the substrate. It was revealed that the surfaces of a composite material based on borosiloxane and ZnO NPs do not inhibit the growth and division of mammalians cells. It is shown in the work that the positive effect of the incorporation of ZnO NPs into borosiloxane can reach 100% or more, provided that the viscoelastic properties of borosiloxane with nanoparticles are retained.

## 1. Introduction

The cytostatic activity of ZnO nanoparticles (NPs) is interesting in relation to tumor cells [1] antimicrobial and fungicidal action [2,3,4], antioxidant and anti-inflammatory actions [5], acceleration of wound healing [6], and antidiabetic properties [7]. From the point of view of medicine and biology, ZnO-based nanoparticles have a number of advantages: high antibacterial efficiency at low concentrations; action on a wide range of bacterial strains, relatively low synthesis cost [8,9,10,11,12]. From a practical point of view, the antibacterial properties of zinc oxides are interesting when added to a material that does not affect the generation or decay rate of long-lived active forms of proteins. Borosiloxane (BS), which has adjustable stickiness [13], the ability to self-repair [14] and dissipation of impact energy [15], can act as such a modern material. The creation of a composition of iron oxides and polymer also solves the problem of particle aggregation due to the fact that iron ions are well distributed along the polymer chain, dispersed over the entire surface [16]. In addition, a polymer with additives of iron oxide NPs usually has hydrophilic surface properties, which helps to prevent the adhesion of microorganisms to the surface [17,18].

BS-based materials are used in various fields. The peculiarity of borosiloxane is that it provides good protection against physical and chemical influences, and also has a low production cost. Another important property of BS is the ability to quickly, like an ordinary liquid, restore integrity when the separated parts are connected, without any traces at the place of rupture. Such ability of BS for self-healing makes it a promising material for various systems with self-healing properties. The use of a composite based on borosiloxane and zinc oxide nanoparticles may be of great interest for use in prostheses and biomedical devices. One of the well-known examples of the use of BS is in sports protective equipment, where materials based on it are used as shock energy absorbers, effectively protecting parts of the human body in such extreme sports as motorcycle and bicycle racing, alpine skiing, etc. Such materials are produced, for example, under the D3O trademark [19]. Thus, the BS system with ZnO NPs with antibacterial properties can be widely used in the production of sportswear.

The areas of biocompatible and self-healing electronics and display technology are relatively new trends for research and development [20,21,22,23,24]. In the previous works of the authors [25,26], new materials based on BS for electro-optical and electronic devices were obtained, investigated, and patented.

## 2. Materials and Methods

### 2.1. Metal Oxide Nanoparticles Synthesis and Characteristics Assay

Metal nanoparticles were obtained by laser ablation in liquid. A pulsed ytterbium-doped fiber laser with a variable pulse width (λ = 1064 nm, τ = 4–200 ns, pulse repetition frequency 20 kHz, average power up to 20 W, pulse energy 1 mJ) was applied. Deionized water in amount of 10 mL was used as a working liquid. A metal target made of the corresponding material (99.9%) was immersed in the liquid so as to provide a liquid layer thickness of 1 mm above the target surface. The irradiation times varied from 5 to 20 min. A detailed description of the setup for the generation of nanoparticles by laser ablation can be found in [27].

A hydrodynamic diameter and zeta potential distribution of nanoparticles were measured with Zetasizer Ultra (Malvern Panalytical, Malvern, UK). The shape, topology of the surface, and element composition of nanoparticles was studied using transmission electron microscope Libra 200 FE HR (Carl Zeiss, Jena, Germany). The spectrum of aqueous colloid of nanoparticles was recorded using an USB3000T (Ocean Optics, Orlando, FL, USA) spectrometer. The extraction of zinc ions from the composite polymer was carried out with deionized water. The concentration of zinc ions was measured using an Ecotest-VA voltammetric analyzer (Moscow, Russia). The sensitivity was 10^−6^ mol/L; the polymer–water ratio was taken as 1/2.

### 2.2. Borosiloxan Composites Synthesis and Rheological Characteristics Assay

The starting materials were hydroxyl-terminated polydimethylsiloxane (PDMS) (molecular weight 20,000 g/mol) (Sigma-Aldrich, Saint Louis, MO, USA) and crushed boric acid (BA) (Sigma-Aldrich, Saint Louis, MO, USA) (basic substance content 99.9%, mass fraction of boric anhydride 57.1%, average particle size BA 0.075 mm). The mass ratio of PDMS and BA was 10 to 1. BS samples were obtained by heating PDMS with BA at a temperature above 200 °C. Borosiloxane was diluted into ethanol and mixed with NPs. Further, ethanol was evaporated at vacuum. Borosiloxane without NPs was also diluted into ethanol and dried. Rheological characteristics of borosiloxane and nanocomposites based on it were measured with modular compact rheometer MCR 302e (Anton Paar, Graz, Austria). To describe the non-Newtonian behavior of systems, one can apply the approach [28], which uses multiparameter rheological equations in a wide range of shear rates.

Study of the effect of composite material on the properties of aqueous solutions.

The composite material was heated to 40 °C and rolled through rollers. After rolling, a polymer film with a thickness of about 700–900 μm was obtained. Rectangular films with a size of 20 × 25 mm were cut from a massive workpiece. The total area of the films is approximately 10 cm^2^ (5 cm^2^ on each side). The films were placed in polypropylene vials and filled with 20 mL of water or aqueous solutions. The methodological subtleties of measuring reactive oxygen species or damage to biological macromolecules are described below.

### 2.3. Measurement of Hydrogen Peroxide Concentration

The highly sensitive method of enhanced chemiluminescence in the luminal, 4-iodophenol, horseradish peroxidase system was used for the quantitative evaluation of hydrogen peroxide concentration in aqueous solutions. Luminescence intensity were measured using ultrasensitive chemiluminometer Biotox-7A-USE (ANO Engineering Center—Ecology, Moscow, Russia). The concentration of the produced hydrogen peroxide was calculated using calibration table (Table 1), which were built on intensity values of chemiluminescence of samples containing known concentration of hydrogen peroxide. The initial H_2_O_2_ concentration used for calibration was evaluated spectrophotometrically with Cintra 4040 (GBC Scientific Equipment, Braeside, Australia) at a wavelength of 240 nm with a molar absorption coefficient of 43.6 M^−1^ × cm^−1^ [29]. The samples were placed in polypropylene vials and 1 mL of a “counting solution” containing 1cM Tris-HCl buffer pH 8.5, 50 μM *p*-iodophenol, 50 μM luminol, and 10 nM horseradish peroxidase were added. The sensitivity of the method makes it possible to determine H_2_O_2_ at a concentration of <1 nM [30].

### 2.4. Measurement of OH-Radicals Concentration

Evaluation of OH-radicals concentration was carried out using the reaction with coumarin-3-carboxylic acid (CCA), which lead to CCA hydroxylation to 7-hydroxycoumarin-3-carboxylic acid (7-OH-CCA). The 7-OH-CCA is a convenient fluorescent probe for determining the formation of these radicals. Experimental samples (and control) were heated in polypropylene vials in a U-10 thermostat (Heizgeräte, Berlin, Germany) at a temperature of 80 ± 0.1 °C for 2 h. The fluorescence of 7-OH-CCA (the product of the reaction of CCA with a hydroxyl radical) was measured on a JASCO 8300 spectrofluorimeter (JASCO, Tokyo, Japan) with λ_ex_ = 400 nm, λ_em_ = 450 nm. Calibration was performed using commercial 7-OH-KKK [29]. Table 2 contains the calibration data used to measure the concentration of 7-OH-KKK in water.

### 2.5. Measurement of Long-Lived Reactive Protein Species Concentration

A chemiluminescence method is an effective and sensitive technic for determining free radical reactions. In this case, the interaction of radicals releases energy emitted in the form of light quanta. The study of long-lived reactive protein species was carried out by measuring the chemiluminescence of protein solutions induced by an increase in temperature using chemiluminometer Biotox-7A (ANO Engineering Center—Ecology, Moscow, Russia). The measurements were performed in the dark, at room temperature, in 20 mL polypropylene vials. The use of large volumes in comparison with standard (0.1 mL) volumes increased the method sensitivity by almost 200 times [30]. All samples were kept in the dark at room temperature for 30 min after exposure. The proteins not exposed to heat served as controls. The method is described in more detail earlier [31,32].

### 2.6. Enzyme-Linked Immunosorbent Assay (ELISA)

A non-competitive enzyme-linked immunosorbent assay (ELISA) with using monoclonal antibodies specific to 8-oxoguanin (anti-8-oxoGua antibodies) was developed for the quantitative measurement of 8-oxoguanine in DNA. DNA samples (350 μg/mL) were denatured by boiling in a water bath for 5 min and cooled in ice for 3–4 min. Aliquots (42 μL) were applied to the bottom of the wells of the ELISA plates. DNA was immobilized using a simple adsorption procedure with incubation for 3 h at 80 °C until the solution was completely dry. Nonspecific adsorption sites were blocked by 300 μL of a solution containing 1% skimmed milk powder in 0.15 M Tris-HCl buffer, pH 8.7, and 0.15 M NaCl. Further, the plates were incubated at room temperature overnight (14–18 h). The antigen-antibody complex formation with anti-8-8-oxoGua antibodies (at a dilution of 1:2000) was carried out in a blocking solution (100 μL/well) by incubation for 3 h at 37 °C. Washed twice (300 μL/well) with 50 mM Tris-HCl buffer (pH 8.7) and 0.15 M NaCl with 0.1% Triton X-100 after 20 min incubation. Further, a complex with conjugate (anti-mouse immunoglobulin labeled with horseradish peroxidase (1:1000)) was formed by incubating for 1.5 h at 37 °C in a blocking solution (80 μL/well). Then, the wells were washed 3 times as described above. Further, a chromogenic substrate containing 18.2 mM ABTS and hydrogen peroxide (2.6 mM) in 75 mM citrate buffer, pH 4.2 (100 μL/well) were added in each well. The reactions were stopped by adding an equal volume of 1.5 mM NaN_3_ in 0.1 M citrate buffer (pH 4.3) upon reaching color. The optical samples density was measured on a plate photometer (Titertek Multiscan, Vantaa, Finland) at λ = 405 nm. The method is described in more detail earlier [33]. Table 3 contains the calibration data used to measure the concentration of 8-oxoGua in DNA. In the calculations, a standard coefficient of 0.78 molecules per 100 eV was used.

### 2.7. Bacteriostatic Activity Assay

Experiments in a cultural environment. Gram-negative bacteria Escherichia coli (LenReactive, St. Petersburg, Russia) were cultured. Using aseptic techniques, we carefully transferred a 5 mL aliquot of LB broth into a sterile, lidded glass culture tube. Using a sterile applicator stick, one well-isolated colony was transferred from the solid medium plate to the culture tube. Then, the colony was resuspended in a glass culture tube. To determine the concentration of bacteria, a spectrophotometric study was carried out. The optical density of the resulting medium was determined using a drop spectrophotometer UV5Nano Excellence (Mettler Toledo, Columbus, OH, USA). For analysis, 10 μL of the medium containing the bacteria was irreversibly taken. After determining the concentration of bacteria, the resulting concentrated medium containing bacteria was diluted in a larger volume. For the experiments, films of a composite material with a thickness of 700–900 μm and a size of 10–15 mm were made. The film was sterilized by three times soaking in ethyl alcohol for 30 min. After that, the film was put on a round sterile hoop. A nutrient medium with bacteria was poured into the hoop, and the top of the hoop was sealed with a piece of glass slide. The resulting structure was placed in an ES-20 incubator shaker (Biosan, Riga, Latvia) (37 °C, approximately 150 rpm). During incubation, the concentration of bacteria was estimated using microscopy and an algorithm developed by us for determining optically dense objects in the frame. At the end of the experiment, the structures were disassembled and the concentration of bacteria was estimated again using a drop spectrometer. 

Experiments on the surface of a solid substrate. Initially, LB agar (Sigma-Aldrich, Saint Louis, MO, USA) was prepared by weighing the appropriate powder medium, agar, and water into a sterile flask. The medium was autoclaved. In a laminar, approximately 25–30 mL of LB agar was poured into sterile Petri dishes. Thereafter, the agar plates were allowed to solidify. Sometimes agar plates were stored upside down at 4 °C for several days. During the experiment, about 100 μL of a suspended culture of *E. coli* was added to a Petri dish with LB agar. The culture was spread over the entire dish using a sterile glass spatula. The Petri dish is incubated for several hours at 37 °C. After that, using a heated composite material, we tried to transfer bacterial cells from the surface of the substrate to the surface of the material. The concentration of microorganisms remaining on the surface was determined using microscopy. Microorganisms stained using a crystal violet indicator and examined under a microscope at 1000 magnification. The details of the experiment were described earlier [34]. Since we used a drop spectrophotometer (optical path length of about 50 μm) and microscopy, we reported the concentration of microorganisms in the number of bacteria per unit area.

### 2.8. Cell Culture

**Figure 1:**
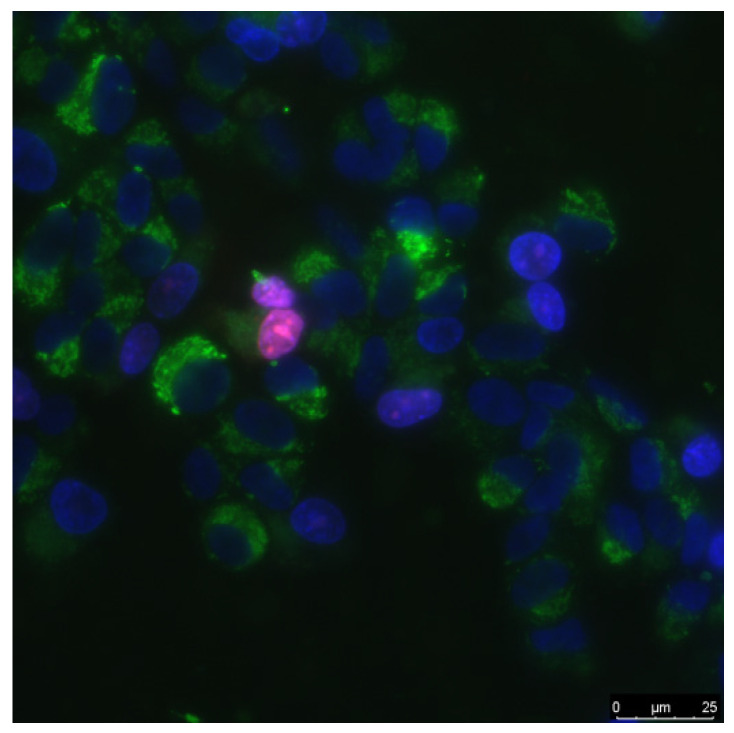
The sample of cell culture micrograph. The mitochondria of the cells are colored green; they can be used to estimate the size of the cells. Normal cell nuclei are colored blue. Nuclei of non-viable cells are stained in purple.

Biocompatibility studies were performed using standard in vitro test systems. Human neuroblastoma SH-SY5Y and mouse primary dermal fibroblasts (Adult, C57BL/6) were cell cultures were used as standard cell models. SH-SY5Y cells in vitro can spontaneously intercontained between two phenotypes, neuroblast-like cells and epithelial-like cells [34]. The cells were grown in DMEM medium (Biolot, Moscow, Russia) supplemented with 10% fetal calf serum (Gibco, Waltham, MO, USA), 30 μg/mL gentamicin, at 37 °C and 5% carbon dioxide in a CO_2_ incubator (Binder, Tuttlingen, Germany). Fragments of material samples 20 × 20 mm in size were placed in Petri dishes with diameter 35 mm, 1 sample pre dish. Then, on the surface of material samples, cells were inoculated at a concentration of 10^4^ cells/cm^2^, in a volume of 3 mL per dish. Cells were cultured on samples during 3 days. Cells growing on the samples surface were stained with fluorescent dyes 2 μg/mL Hoechst 33342 (Sigma-Aldrich, Saint Louis, MO, USA) and 2 μg/mL propidium iodide (Sigma-Aldrich, Saint Louis, MO, USA) to determine the numbers of living and dead cells, respectively. Hoechst 33342 stains all cells (live and dead). The propidium iodide dye penetrates into living cells extremely slowly, therefore, during the short incubation time (we used about 10 min), it stains only cells with a damaged plasma membrane. The plasma membrane with breaks leading to dye penetrates was one of the main criterion that the cell is dead. Thus, Hoechst 33342 stains both living and dead cells, while propidium iodide only stains dead cells (Figure 1). Microscopic assay of the samples was carried out with imaging system based on Leica DMI6000 (Leica, Berlin, Germany). On the surface of each sample at least 500 cells were counted for analysis [35].

The mitotic index of cells in the logarithmic growth phase (3 days from the moment of seeding) was used to analyze proliferation of cells. The number of cells in a state of mitosis was determined using fluorescence microscopy using in vitro staining with the Hoechst 33342 fluorescent dye (Sigma-Aldrich, Saint Louis, MO, USA). Mitotic cells were identified by the chromatin distribution characteristic of prophase (P), metaphase (M), anaphase (A), and telophase (T). For analysis, at least 500 cells were counted on the each sample surface. The mitotic index (MI) was calculated by the formula MI = (P + M + A + T)/N × 100%, where (P + M + A + T) is the number of cells at the stage of prophase, metaphase, anaphase, and telophase, respectively, and N is the total number of analyzed cells [36].

### 2.9. Determination of Changes in Gene Expression

Gene expression was analyzed in the mouse primary dermal fibroblasts cells by RT PCR in real time after exposure to composite material. The genes encoding of enzymes of antioxidant response CAT, SOD1, and transcription factors NRF2 were analyzed. The procedure for isolation of total RNA, cDNA synthesis, and PCR arrangement in real time is described in detail in [23]. The obtained cDNA was subsequently used in PCR with gene-specific primers (Table 4) synthesized by “Evrogen” (Moscow, Russia). To compare the levels of gene expression we used REST 2005 software (Qiagen, Hilden, Germany) (version 1.9.12).

### 2.10. Statistic

The data were analyzed using GraphPad Prism eight (GraphPad Software, San Diego, CA, USA) and Origin software (OriginLab Corporation, Northampton, MA, USA) and were presented as means ± SEM. Data from at least three independent experiments were used for averaging.

## 3. Results

### 3.1. Physicochemical Characteristics of Materials and Composite

**Figure 2:**
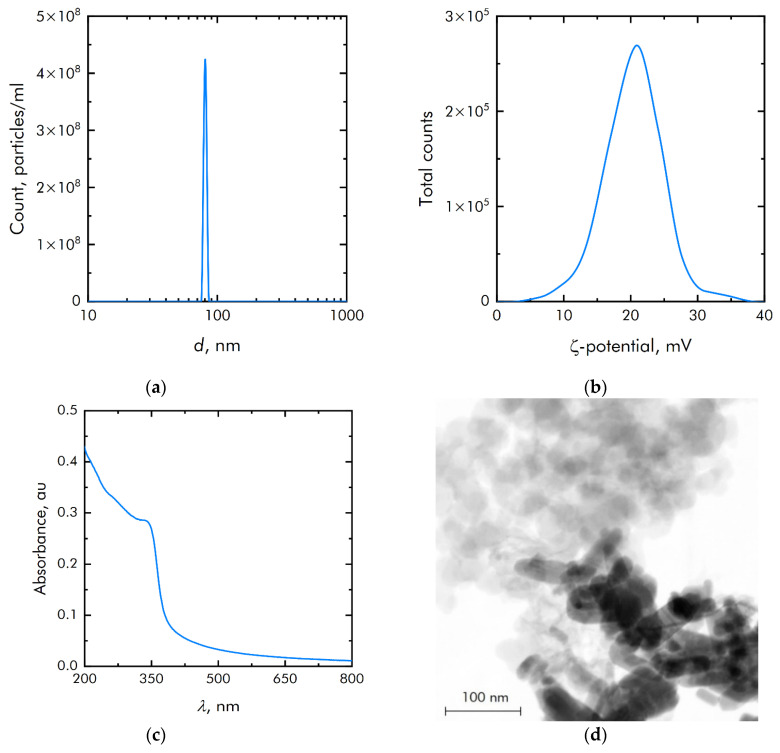
Physicochemical properties of zinc oxide nanoparticles: (**a**) Concentration and size distribution of zinc oxide nanoparticles; (**b**) Zeta potential of zinc oxide nanoparticles; (**c**) Optical absorption of an aqueous colloidal solution of zinc oxide nanoparticles; (**d**) TEM image of a group of zinc oxide nanoparticles (image used by the authors earlier in other manuscripts).

ZnO NPs were obtained by laser ablation in water. The ZnO NPs size distribution was unimodal and rather narrow (Figure 2a). The average hydrodynamic diameter of ZnO NPs was about 90 nm. The concentration of NPs was of the order of 4.3 × 10^8^ NPs/mL. The zeta potential distribution of NPs was unimodal (Figure 2b). The zeta potential of NPs was distributed from +7 to +35 mV. The average zeta potential was +20.6 mV. Figure 2c shows the absorption spectrum of an aqueous colloidal solution of ZnO NPs. It is shown that the absorption spectrum corresponds to the spectrum of ZnO NPs. The electron microscopy of the obtained samples was carried out to establish elemental analysis and independently confirm the size of NPs. The NPs have been shown to be on the order of 90 nm (Figure 2d). They are composed of the chemical elements zinc and oxygen. The ratio of atoms Zn/O = 1, respectively, therefore NPs mainly consist of ZnO.

Investigated the extraction of zinc ions from the composite polymer (Table 5). Extraction was carried out with deionized water. Approximately 10 g of composite film was filled with 20 mL of deionized water. Extraction took place at room temperature. During the first 10 days of the experiment, the concentration of zinc ions in the eluent was less than 1 μM (close to the sensor’s sensitivity limit). After 20 days, the concentration of ions increased to 3 μM, and then rapidly increased over the next 25 days. Then, 45 days after the start of the experiment, the rate of transition of zinc ions from the composite material to the solution decreased significantly. During the experiment, less than 10% of all zinc ions from composite material were introduced into the solution.

**Figure 3:**
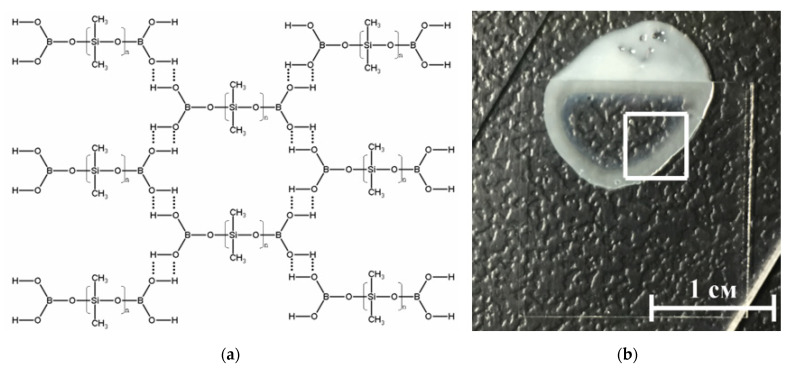
(**a**) Schematic representation of the structure of the borosiloxane (BS). BS molecules linked through hydrogen bonds; (**b**) Photography of composite material, part of the sample is under the cover glass.

BS-based material was chosen as a matrix of nanoparticles (Figure 3). From the point of view of the molecular structure, BSs belong to the class of organosilicon compounds containing the R-Si-O-B group, where R is a hydrocarbon radical.

Depending on the molecular structure and molecular weight of the initial organosiloxane, the temperature regimes of BS synthesis, as well as on the amount and properties of the introduced functional additives (fillers, plasticizers, thickeners, etc.), BS-based materials can have different mechanical properties. They can be in both solid and liquid state of aggregation, and also have a viscous, rubbery, or glassy consistency [37]. Highly elastic behavior is realized due to stretching of siloxane fragments, and viscous due to the fact that these formed layers can freely move relative to each other. Thus, brittle fracture of BS and composites based on it can occur many times without changing the characteristics of the material and the molecular weight of its constituent molecules, since there is no destruction of covalent bonds.

**Figure 4:**
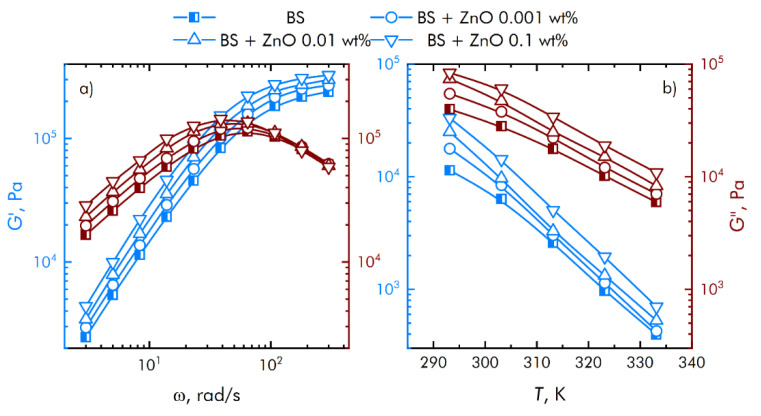
Influence of composite material based on boroxiloxane and zinc oxide nanoparticles on the mechanical spectra; storage modulus—blue symbol, loss modulus—red symbol. (**a**) The dependence of the change in the mechanical spectra of composites on the rotation speed. (**b**) Temperature dependence of the change in the mechanical spectra of composites.

Assessment of the rheological characteristics of BS and systems based on it is important for the possibility of their further application in bioactive substances. Figure 4 shows the concentration dependences of the real and imaginary parts of the dynamic elastic modulus at different concentrations of zinc oxide NPs. With an increase in the concentration of zinc oxide, an increase in the viscoelastic properties is observed. BS has complex rheological responses when shear loads are applied due to microstructural reorganization.

Based on obtained data (Figure 4), we concluded that at low shear rates, the prevalence of the viscous properties of the BS over the elastic ones is observed, with an increase in the speed, the contribution of the viscous properties decreases, while the storage modulus reaches a constant value. In this case, the region of transition from viscous to elastic properties is adjusted at the stage of BS synthesis, depending on the amount and properties of the introduced functional additives. This behavior of borosiloxane significantly distinguishes it from other polymer matrices [38,39,40] for nanoparticles.

### 3.2. Influence of Composite on ROS Generation and Damage to Biomolecules

**Figure 5:**
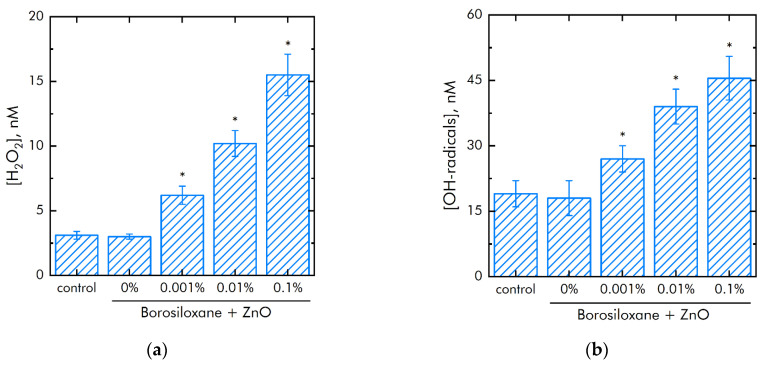
Effect of composite material containing borosiloxane and ZnO nanoparticles on the generation of reactive oxygen species: (**a**) Formation of hydrogen peroxide (2 h, 40 °C); (**b**) Generation of hydroxyl radicals (2 h, 80 °C); * indicate a significant difference at 5% level in comparison with the control (*p* < 0.05). Data are presented as mean values and standard errors.

The metals of variable valence are capable to generate of reactive oxygen species (ROS). The effect of polymer and ZnO NPs on the generation of reactive oxygen species (hydrogen peroxide, as the most stable representative (Figure 5a) and hydroxyl radicals, as the most reactive (Figure 5b)) was studied. It was shown that borosiloxane has no effect on the generation of hydrogen peroxide. A composite material based on borosiloxane and ZnO nanoparticles doubles the rate of hydrogen peroxide generation at a nanoparticle mass concentration of 0.001%.

The rate of generation of hydrogen peroxide increases in 5 times with an increase in the mass concentration of ZnO NPs. The borosiloxane has no effect on the generation of hydroxyl radicals. In this case, a composite material based on borosiloxane and zinc oxide nanoparticles increases the rate of generation of hydroxyl radicals about 1.5, 2, and 2.5 times at a ZnO NPs concentration of 0.001%, 0.01%, and 0.1%, respectively.

**Figure 6:**
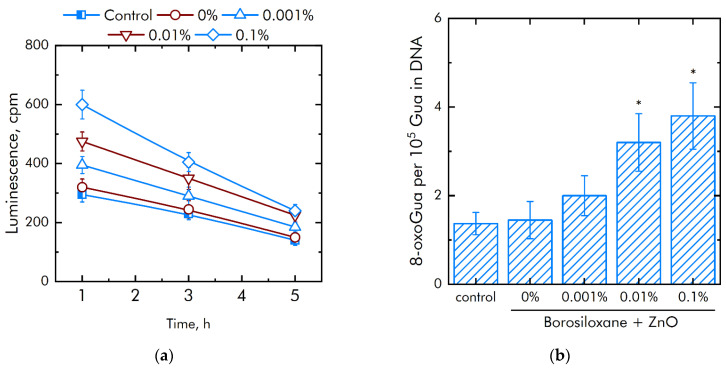
Effect of composite material containing borosiloxane and ZnO nanoparticles on the generation on biomacromolecules damage: (**a**) Formation of long-lived reactive protein species (2 h, 40 °C); (**b**) Generation of 8-oxoguanosine (2 h, 45 °C); * indicate a significant difference at 5% level in comparison with the control (*p* < 0.05). Data are presented as mean values and standard errors.

It is known that excess ROS generation is associated with damage to biomacromolecules. The effect of a composite material based on borosiloxane and ZnO NPs on the formation of long-lived active forms of proteins was studied (Figure 6a). It was shown that upon contact with borosiloxane, which does not contain ZnO NPc, the rate of formation and decomposition of long-lived active forms of proteins does not differ from the control. Addition of ZnO NPs in the polymer significantly increased rate of generation of long-lived active forms of proteins. An increase in the rate by 23%, 1.5 times and 2 times is observed at a concentration of ZnO NPs 0.001, 0.01 and 0.1%, respectively. At the same time, ZnO NPs have almost no effect on the average half-life of long-lived active forms of proteins. The half-life is about 4–5 h in all experimental groups.

The effect of a composite material based on borosiloxane and ZnO NPs on the formation of 8-oxoguanine in DNA in vitro was studied (Figure 6b). It was found that borosiloxane did not affect the formation of 8-oxoguanine in DNA in vitro. The rate of 8-oxoguanine formation in DNA significantly increases when ZnO NPs appear in the polymer. Addition of ZnO NPs in concentration 0.01% and 0.1% increased rate of 8-oxoguanine formation in DNA in 2 and 2.3 times, respectively.

### 3.3. Influence of the Composite on the Growth and Development of Eukaryotic and Prokaryotic Cells

**Figure 7:**
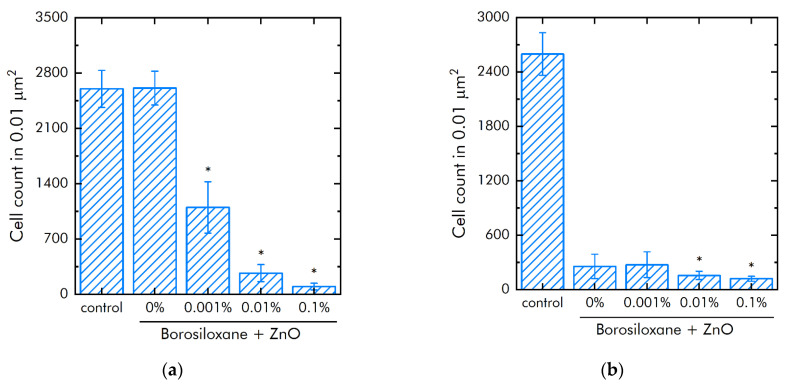
Influence of composite material based on borosiloxane and zinc oxide nanoparticles on the growth and development of *E. coli*: (**a**) Development of *Escherichia coli*. Incubation time is 24 h; (**b**) Effect of tearing off bacteria from a substrate using a composite material based on borosiloxane and zinc oxide nanoparticles; * indicate a significant difference at 5% level in comparison with the control (*p* < 0.05). Data are presented as mean values and standard errors.

The effect of ZnO NPs in borosiloxane on both the growth and development of bacteria *E. coli* was studied (Figure 7a). Borosiloxane without NPs did not affect the growth and development of *E. coli*. Addition of ZnO NPs in the polymer decreased the density of bacterial cultures grown on the composite by 58%, 90%, and 96% at a nanoparticle concentration of 0.001%, 0.01%, and 0.1%, respectively. In a separate series of experiments, the bacteriostatic properties of an aqueous colloidal solution of zinc oxide nanoparticles were investigated. The concentrations studied were 0.0001%, 0.001%, 0.01%, and 0.1%. It was shown that at nanoparticle concentrations of 0.001%, 0.01%, and 0.1%, the growth and development of bacteria is not observed. At a concentration of 0.0001%, the density of the bacterial culture is 93% less compared to the control.

The effect of a composite material based on borosiloxane and ZnO NPs on the detachment of *E. coli* from the substrate was investigated (Figure 7b). Borosiloxane without ZnO NPs effectively detached the *E. coli* bacteria from the substrate. The number of bacteria on the substrate decreases in about 10 times. The addition of ZnO NPs to the polymer at a mass concentration of 0.001% and 0.01% has no significant effect. With an increase in the concentration of NPs in the polymer to 0.1%, the detachment of bacteria from the substrate occurs 2 times more efficiently as compared to pure BS. The number of bacteria on the substrate is reduced in 22 times.

**Figure 8:**
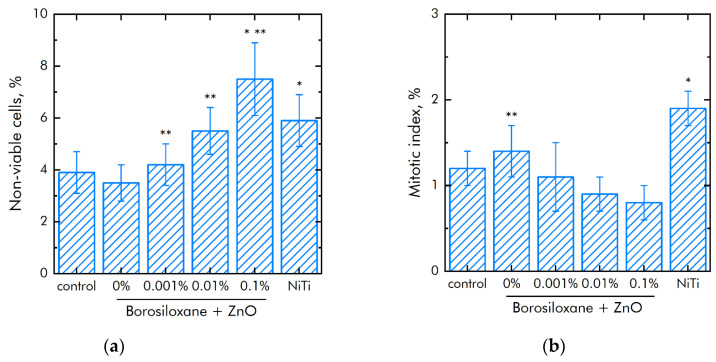

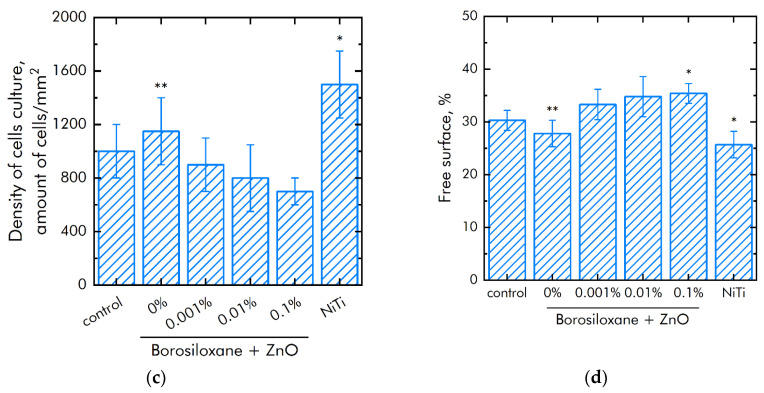
Effect of composite material based on borosiloxane and zinc oxide nanoparticles on the main characteristics of cell culture SH-SY5Y growth and development: (**a**) Cell viability; (**b**) Mitotic index of a cell population; (**c**) Cell culture density; (**d**) Colonization rate; * indicate a significant difference at 5% level in comparison with the control (*p* < 0.05). ** indicate a significant difference at 5% level in comparison with the NiTi group (*p* < 0.05). Data are presented as mean values and standard errors.

The effect of a composite material based on borosiloxane and ZnO NPs on the viability of mammalian cells was studied (Figure 8a). The number of non-viable cells grown on control substrates and culture plastic did not exceed 4%. Approximately the same number of non-viable cells was observed when grown on a composite material based on borosiloxane without ZnO NPs or contains 0.001% nanoparticles. At the same time, when nitinol is used as a substrate, the number of non-viable cells was almost 50% higher (about 6%). Proportions of non-viable cells were about 5.5% and 7.5% at 0.01% and 0.1% ZnO NPs concentrations in nanocomposite, respectively.

The mitotic index of cells in the logarithmic growth phase was used to analyze the ability of cells to divide (Figure 8b). It was found that the mitotic index of the culture of cells growing on the surface of the titanium sample is 1.2%. The mitotic index is 1.9% when nitinol is used as a substrate. The mitotic index was 0.8–1.1% when cells grown on a composite material based on borosiloxane and ZnO NPs nanoparticles.

The cell culture density grown on titanium was averages 1050 cells/mm^2^ (Figure 8c). The density of cells grown on a nitinol substrate reaches was 1450 cells/mm^2^. The cells density grown on a composite material based on borosiloxane and ZnO NPs reaches 700–900 cells/mm^2^. Morphological analysis was carried out after 72 h of culturing the cells on the surface of the materials. It was found that the surfaces of a composite material based on BS ZnO NPs are more suitable for cell attachment and spreading (Figure 8d). Moreover, the degree of suitability is comparable to culture plastic and the medical alloy nitinol. At the same time, after 72 h of culturing on the surface of all samples of materials, the cells did not form a 100% monolayer. Only some of its elements are observed. On the surface of all materials, cells occupied about 70–75% of the surface available for growth.

Table 6 shows the effect of a colloidal solution of zinc oxide nanoparticles on the growth and development of mammalian cells. In these experiments, the cells were in contact only with zinc oxide nanoparticles and not in contact with the polymer. In this series of experiments, the nanoparticles were in the bulk, and not on the polymer surface. It is shown that nanoparticles have an effect on the number of viable cells and the value of the mitotic index at significantly lower concentrations compared to a polymer containing zinc oxide nanoparticles.

**Figure 9:**
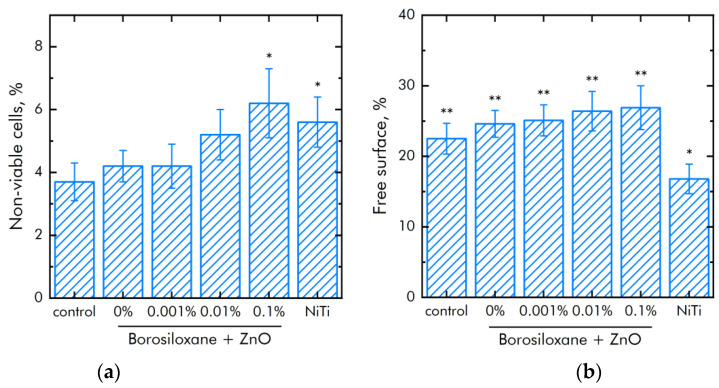
Effect of composite material based on borosiloxane and zinc oxide nanoparticles on the main characteristics of mouse primary dermal fibroblasts cell culture growth and development: (**a**) Cell viability; (**b**) Colonization rate; * indicate a significant difference at 5% level in comparison with the control (*p* < 0.05). ** indicate a significant difference at 5% level in comparison with the NiTi group (*p* < 0.05). Data are presented as mean values and standard errors.

The effect of composite polymer on the viability of mouse primary dermal fibroblasts cells was studied (Figure 9a). It was found that the number of non-viable cells on control and polymer coatings without NPs are about 4%. On polymers with NPs 0.001% and 0.01%, the number of non-viable cells did not differ significantly compared to control. On the polymer with a nanoparticle concentration of 0.1%, slightly more non-viable cells were observed compared to the control. However, the number of non-viable cells on the polymer containing 0.1% NPs of zinc oxide did not differ from those on the medical alloy nitinol. All materials used in the work did not have a short-term toxic effect on mouse primary dermal fibroblasts cells. Figure 9b shows the effect of polymer with zinc oxide nanoparticles on the degree of population of accessible surfaces with cells. By the third day of culturing cells on polymer coatings that do not contain NPs, approximately 20% of the surface remains uninhabited. With the addition of NPs, there is a tendency to less quickly populate surfaces.

The expression of a number of genes in mouse primary dermal fibroblasts cells grown in the presence of different substrates has been investigated. The expression of genes for the antioxidant enzymes catalase (CAT) and superoxide dismutase 1 (SOD1), as well as the expression of the transcription factor NRF2, which is responsible for the response of the cell genome to stressful situations, has been studied (Table 7). It was shown that in the presence of borosiloxane, the expression of SOD1 increases by a quarter and the expression of NRF2 decreases by a quarter. When zinc oxide nanoparticles are added to the polymer, the expression of all studied genes increases. There is a tendency towards concentration-dependent increase. In cells growing on a composite material with a nanoparticle concentration of 0.1%, the expression level is slightly higher than in cells growing on nitinol.

## 4. Discussion

An important mechanism of materials nanotoxicity is the formation of reactive oxygen species (ROS). Overproduction of ROS can cause oxidative stress, as a result of which cells cannot maintain normal physiological redox functions [41]. In particular, ZnO NPs cause apoptosis of cells of the human leukemia line HL60 and death of gram-positive bacteria through the induction of lipid peroxidation [42].

ROS play a useful physiological role in cellular signaling systems and in the induction of mitogenic responses [43,44]. It was found from Figure 5 that the material developed by the BS does not have any effect on the generation of oxygen. At the same time, some types of polymer matrix [45,46] are involved in oxygen production. At the same time, as shown in [38], additives to the polymer matrix impair the mechanical properties of hydrogels; in our case (Figure 3), the mechanical properties practically do not depend on the mass content of nanoparticles, and strict control of the viscoelastic properties of composites for various tasks is carried out even at polymer synthesis stage. As a rule, damage to cell function and development includes oxidative modification of proteins for the generation of protein radicals [47], initiation of lipid peroxidation [48,49]. The presented composite material increases the rate of hydrogen peroxide generation almost 2 and 5 times at a mass concentration of nanoparticles of 0.001–0.1 a concentration of ZnO NPs. This behavior of ZnO in redox reactions leading to the formation of ROS correlates well with the works [50,51]. It was found that borosiloxane has no effect on the generation of hydroxyl radicals (Figure 4b). In this case, a composite material based on borosiloxane and zinc oxide nanoparticles increases the rate of generation of hydroxyl in 1.5–2.5 times, which significantly distinguishes the results obtained from other works [52,53].

As a rule, polymeric materials resistant to the formation of 8-oxoguanine in DNA in vitro are of great interest in medicine [54], because 8-oxoguanine is key biomarker of oxidative DNA damage. 8-oxoguanine has the properties of ambiguous coding and can lead to the formation of mismatched nucleotides with adenine, which, in turn, makes GC-TA transversion possible. In mammals, there are at least four complex mechanisms for removing 8-oxoguanine from DNA and for preventing its incorporation into DNA. The presence of such a number of duplicating mechanisms suggests that the cell perceives 8-oxoguanine as an extremely serious threat that must be quickly eliminated [55]. Studies (Figure 5b) show that borosiloxane did not affect the formation of 8-oxoguanine in DNA in vitro, which significantly distinguishes it from other materials [56,57,58]. It was shown that when zinc oxide nanoparticles appear in BS, the rate of 8-oxoguanine formation in DNA significantly increases. At a concentration of zinc oxide nanoparticles of 0.01% and 0.1% the rate of 8-oxoguanine formation in DNA increases in 2 and 2.3 times, respectively.

One of the main causes of infections on the surface of a biomaterial is the biomaterial itself [59,60]. Postoperative contamination with airborne microorganisms and skin flora remains the most common pathway for contamination of medical devices [61,62]. Microorganisms adhere to the surfaces of the biomaterial and grow, forming biofilms. We found (Figure 7a) that borosiloxane, which does not contain nanoparticles of zinc oxide, did not affect the growth and development of *E. coli* bacteria. In this case, when ZnO NPs appear in the polymer, the density of bacterial cultures grown over the composite decreases by 58% at a nanoparticle concentration of 0.001%, by 90% at a nanoparticle concentration of 0.01%, and by 96% at a nanoparticle concentration of 0.1%. Such composites [63,64,65] possess high disinfecting properties not only against *E. coli* bacteria, but also against bacteria *S. aureus*, *K. pneumonia*, *L. monocytogenes*, and *B. subtilis*. However, nanoparticle concentrations often start from 1 wt%, and the antibacterial efficacy of the resulting composites is lower than in Figure 6. We found that borosiloxane, which does not contain zinc oxide nanoparticles (Figure 7b), is able to effectively detach *E. coli* bacteria from the substrate. The number of bacteria on the substrate decreases by one order of magnitude. With an increase in the concentration of nanoparticles in the polymer to 0.1%, the detachment of bacteria from the substrate occurs 2 times more efficiently as compared to pure borosiloxane.

Modern techniques for manipulating living cells using nanocoatings [66] are associated with understanding the interactions between the polymer-nanoparticle system with mammalian cells [67,68]. As a rule, PEG poly (ethylene glycol), which is biocompatible in vivo, is used to modify nanostructures and deliver drugs [69,70]. At the same time, PEG does not have the ability to self-repair and dissipate the impact energy of biomedical devices. Another important property of BS is the ability to quickly, like an ordinary liquid, restore integrity when the separated parts are connected, without any traces at the place of rupture. The studies of the BS system of zinc oxide nanoparticles (Figure 8a) on non-viable cells grown on control substrates and culture plastic did not exceed 4%. Approximately the same number of non-viable cells were observed when grown on a composite material based on borosiloxane, which does not contain zinc oxide nanoparticles or contains 0.001% nanoparticles. At the same time, when nitinol is used as a substrate for the medical alloy, an almost 50% greater number of nonviable cells is observed (approximately 6%). Comparison of the density of cell cultures is 1050 cells/mm^2^—titanium, 1450 cells/mm^2^—nitinol, 700–900 cells/mm^2^—BS of zinc oxide nanoparticles. Thus, the obtained BS system of zinc oxide nanoparticles possesses antibacterial properties at low concentrations of nanoparticles and retains its viscoelastic characteristics.

## 5. Conclusions

Borosiloxane provides good protection against physical and chemical influences on nanoparticles and has a low production cost. The resulting composite based on borosiloxane and zinc oxide nanoparticles is of great interest for use in prostheses and biomedical devices. A significant increase in bacteria detachment, together with antibacterial properties, makes the developed material especially attractive for use as a reusable dry disinfectant. It is important that the developed composite material does not affect the viability and development of mammalian cells. Thus, the resulting biocompatible media, in terms of their rheological characteristics, can be the basis for the creation of antibacterial systems.

## Figures and Tables

**Table 1:** Calibration data used to determine the concentration of hydrogen peroxide in aqueous solutions (data from one of the experiments).

H_2_O_2_ Concentration Added to the Sample, nM	Luminescence Intensity, cps
0	52
2	851
5	2076
10	4092
15	6121
20	81452

**Table 2:** Calibration data used to determine the concentration of 7-OH-KKK in aqueous solutions (data from one of the experiments).

7-OH-KKK Concentration Added to the Sample, nM	Fluorescence Intensity, a.u.
0	0.1
5	2.2
10	4.2
20	8.3
30	12.1
40	16.2

**Table 3:** Calibration data used to determine the concentration of 8-oxoGua in DNA (data from one of the experiments).

Dose, Gy	8-oxoGua per 10^5^ Gua in DNA
0	0.01
1	0.78
2	1.56
5	3.90
10	7.80

**Table 4:** Oligonucleotides used for qRT-PCR. The design of oligonucleotides was carried out with the use of Primer-BLAST (www.ncbi.nlm.nih.gov) (10 August 2021). The calculated Tm for all primers is 61–63 °C. Real-time PCR was performed at Tm = 60 °C. PCR products were melted from 60 °C to 90 °C to assess the specificity of the reaction.

Genes	GenBank Accsession	Oligonucleotides 5′-3′ (F+R)	Amplicon Size, bp
Actb	NM_007393.4	CCTTCCTTCTTGGGTATGGAATCC CACCAGACAGCACTGTGTTGGCA	115
CAT	NM_009804	AGCGACCAGATGAAGCAGTG TCCGCTCTCTGTCAAAGTGTG	181
SOD1	NM_011434	AACCAGTTGTGTTGTCAGGAC CCACCATGTTTCTTAGAGTGAGG	139
NRF2	NM_010902	CTCGCTGGAAAAAGAAGTG CCGTCCAGGAGTTCAGAGG	240

**Table 5:** Effect of extraction time on the release of Zn^+^ from a polymer.

Extraction Time, Days	Concentration of Zn^+^ in Solution, μM
0	>1
5	>1
10	>1
15	1
20	3
25	7
30	9
45	11
60	12

**Table 6:** The effect of a colloidal solution of zinc oxide nanoparticles on the growth and development of cells.

Parameter	Concentration of Nanoparticles, %			
	Control	0.0001	0.001	0.01
Viable cells, %	97.08 ± 1.59	96.03 ± 1.97	95.86 ± 1.49	93.78 ± 1.77
Mitotic index, %	1.22 ± 0.37	1.24 ± 0.35	1.02 ± 0.23	0.74 ± 0.26
Average cell area, μm^2^	135.33 ± 24.24	146.71 ± 24.74	124.78 ± 22.05	132.38 ± 26.95

**Table 7:** Changes in the expression of particular genes in mouse primary dermal fibroblasts cells.

Genes	Level of mRNA Relative to bAct = 1	Change The Level of Gene Expression (Fold) Relative to 0 Gy (-)					
		Control	Borosiloxane + Zn (%)				NiTi
			0%	0.001%	0.01%	0.1%	
CAT	3.7 × 10^−3^	1	0.98	1.08	1.89	3.39	2.31
SOD1	3.6 × 10^−2^	1	1.24	1.41	1.13	2.06	1.79
NRF2	2.2 × 10^−3^	1	0.75	1.26	2.04	2.15	1.96

## Data Availability

The raw data supporting the conclusions of this article will be made available by the authors, without undue reservation.
